# Genome-Wide Identification and Characterization of the *14-3-3* Gene Family in *Avena sativa*

**DOI:** 10.3390/plants15091280

**Published:** 2026-04-22

**Authors:** Shirui Xu, Mingchuan Ma, Zhang Liu, Lijun Zhang, Longlong Liu

**Affiliations:** 1 Center for Agricultural Genetic Resources Research, Shanxi Agricultural University, Taiyuan 030031, China; 2 Houji Laboratory in Shanxi Province, Center for Agricultural Genetic Resources Research, Shanxi Agricultural University, Taiyuan 030031, China; 3 Key Laboratory of Minor Crop Germplasm Innovation and Molecular Breeding (Co-Construction by Ministry and Province), Ministry of Agriculture and Rural Affairs, Taiyuan 030031, China

**Keywords:** *Avena sativa*, genome-wide identification, *14-3-3* gene family, expression pattern, drought stress

## Abstract

14-3-3 proteins are highly conserved regulatory proteins that integrate signaling pathways governing plant growth, development, and stress responses. However, the *14-3-3* gene family in oat (*Avena sativa*) has not been systematically investigated. Here, we performed a comprehensive analysis of oat *14-3-3* genes, including their physicochemical properties, gene structures, phylogeny, conserved motifs, promoter *cis*-elements, and selective pressures. A total of 19 *AsGF14* genes were identified and classified into the ε and non-ε groups. The *AsGF14* gene family expanded primarily through segmental duplications and has been under strong purifying selection during evolution. qRT-PCR analysis revealed that six *AsGF14* genes were significantly upregulated at one or more time points under drought stress. Notably, *AsGF14k* exhibited sustained and significant upregulation. Subcellular localization analysis showed that AsGF14k localized to both the nucleus and the cytoplasm. Furthermore, Y2H assays indicated that AsGF14k does not form homodimers. Our results provide a systematic characterization of the *AsGF14* gene family and their drought-responsive expression patterns, establishing a preliminary basis for the functional validation of *AsGF14* genes under drought stress.

## 1. Introduction

14-3-3 proteins are a family of highly conserved, small, acidic regulatory proteins ubiquitously expressed in all eukaryotes [1]. First discovered in bovine brain approximately 60 years ago, these proteins were subsequently identified in plants about 30 years later [2,3]. In plants, they are designated as GRF (General Regulatory Factor) or GF14 (G-box Factor 14-3-3) owing to their role as components of the G-box protein complex. 14-3-3 proteins function primarily as homodimers or heterodimers [4,5]. Although lacking intrinsic enzymatic activity, they specifically recognize phosphorylated serine/threonine motifs on target proteins, thereby regulating their subcellular localization, stability, and activity [6,7,8]. Through this mechanism, 14-3-3 proteins play pivotal roles in signal transduction and metabolic regulation. Phylogenetic analyses classify plant 14-3-3 isoforms into two major groups: the ε group and the non-ε group [9]. Notably, non-ε isoforms exhibit both stronger binding to and more potent activation of plasma membrane H^+^-ATPase than ε isoforms, as demonstrated in *Arabidopsis* [10]. Plants comprise multiple cell types, and developmental and stress responses arise from complex intercellular interactions [11]. Given this complexity, protein localization at the cell-type level is essential for understanding functional mechanisms. 14-3-3 proteins localize to various subcellular compartments (e.g., nucleus, plasma membrane, cytoplasm, and mitochondria), mediating diverse interactions with target proteins and distinct functional roles [4,12]. Notably, the same 14-3-3 protein can display different localization patterns across cell types, indicating cell-type-specific functionality [13].

As pivotal regulatory hubs in plants, 14-3-3 proteins integrate diverse signaling pathways that govern essential processes, including growth, development, and responses to abiotic and biotic stresses [6,14,15]. The conserved and multifunctional roles of these proteins have been elucidated across various species. For instance, in *Arabidopsis*, 14-3-3λ and κ facilitate the assembly of the phyB-PIF3-PPK complex to modulate light signaling [16]. Similarly, heterologous overexpression of mango *MiGF6A* and *MiGF6B* in *Arabidopsis* accelerates flowering [17]. In soybean, the 14-3-3 protein GmSMS6 is a key regulator of seed development, influencing both seed weight and protein content [18]. Beyond developmental roles, 14-3-3 proteins are central to stress adaptation. The MdPBL34-MdGRF10 phosphorylation module stabilizes MdASMT1 to confer salt tolerance in apple [19]. Additionally, interactions between At14-3-3PSI/Nt14-3-3C and the metabolic enzymes AtMDH1/AtGS1 enhance formaldehyde detoxification [20]. In rice, OsGF14d acts as a positive regulator of cold stress tolerance and is targeted for mono-ubiquitination by OsATL38 [21].

Given their extensive involvement in critical signaling pathways, the *14-3-3* gene family has been systematically characterized in several major cereal crops. To date, genome-wide analyses have identified 17, 8, 28, 6, 6, and 8 *14-3-3* genes in wheat [22], rice [12], maize [23], sorghum [23], barley [24], and foxtail millet [25], respectively. In contrast, the *14-3-3* gene family in oat has not yet been comprehensively characterized, despite its agricultural and nutritional importance.

Oat (*Avena sativa* L.) is an allohexaploid cereal ranked seventh in global production and valued for its diverse array of health-promoting compounds [26,27], such as β-glucan, phenolic acids, tocols, sterols, avenacosides, avenanthramides, as well as unique proteins and peptides [28,29,30]. In China, oat is widely cultivated on arid and semi-arid marginal lands in northern and northwestern regions, where drought stress constitutes a primary constraint on growth and yield potential [31,32]. To identify candidate *14-3-3* genes potentially involved in oat drought response, this study aimed to (1) systematically characterize the *14-3-3* gene family in oat genome-wide and (2) profile their drought-responsive expression patterns. This work provides a basis for future functional studies of the *14-3-3* gene family in oat drought adaptation.

## 2. Results

### 2.1. Identification, Characterization, and Chromosomal Localization of AsGF14 Genes in A. sativa

In this study, 19 *AsGF14* genes were identified in the oat genome and designated *AsGF14a* to *AsGF14s* based on their chromosomal locations (Table 1, Table S1). The deduced AsGF14 proteins ranged from 248 to 266 amino acids in length, with molecular weights between 28.17 and 29.67 kDa. Their theoretical isoelectric points (pI) varied from 4.66 to 5.10, with an average of 4.81. Thirteen of the 19 AsGF14 proteins exhibited instability indices above 40, indicating they are likely unstable; only AsGF14d, AsGF14g, AsGF14i, AsGF14k, AsGF14o, and AsGF14s showed values below this threshold. Additionally, all AsGF14 proteins exhibited negative grand average of hydropathy (GRAVY) values, confirming their hydrophilic nature. Multiple sequence alignment revealed that the N- and C-terminal regions are poorly conserved, whereas the central region is relatively conserved among AsGF14 proteins (Figure S1). 3D structure prediction of AsGF14 proteins revealed a conserved core region composed of nine α-helices, with α3 and α4 being the longest helices across all AsGF14 proteins (Figure S2).

**Figure 1:**
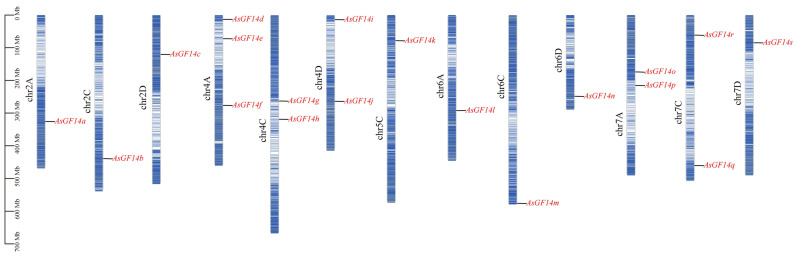
Chromosome localization of *AsGF14* genes.

Chromosomal localization analysis revealed that the 19 *AsGF14* genes were distributed across 13 oat chromosomes belonging to homoeologous groups 2, 4, 5, 6, and 7 (Figure 1). The number of *AsGF14* genes assigned to each homoeologous group ranged from 1 to 7. Each chromosome harbored one to three *AsGF14* genes: a single gene was located on chromosomes 2A, 2C, 2D, 5C, 6A, 6C, 6D, and 7D, whereas three genes (*AsGF14d*, *AsGF14e*, and *AsGF14f*) were clustered on chromosome 4A. In total, the A and C sub-genomes each contained seven *AsGF14* genes, and the D sub-genome carried five.

### 2.2. Phylogenetic and Synteny Analyses

**Figure 2:**
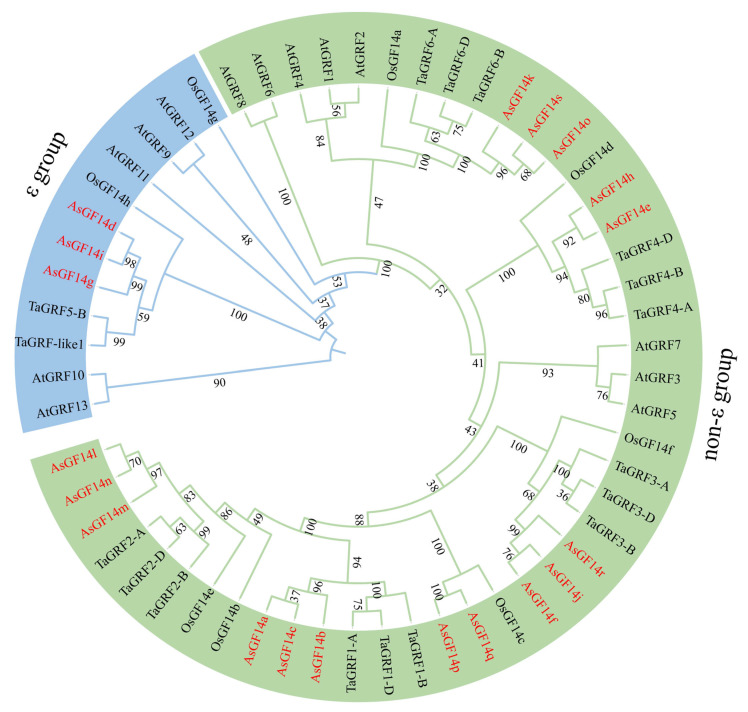
Phylogenetic analysis of *14-3-3* gene family members in *A. sativa*, *A. thaliana*, *O. sativa* and *T. aestivum*. Different groups are indicated by different colors.

To investigate the evolutionary relationships within the *14-3-3* gene family, a phylogenetic tree was constructed using protein sequences from *A. sativa*, *A. thaliana*, rice (*Oryza sativa*), and wheat (*Triticum aestivum*) (Figure 2). Based on sequence similarity, the 14-3-3 proteins were clearly classified into two distinct groups: the ε group and the non-ε group. The ε group comprises 12 members, including three from oat, five from *A. thaliana*, two from rice, and two from wheat. In contrast, the non-ε group contains 45 members: 16 from oat, eight from *A. thaliana*, six from rice, and 15 from wheat. Notably, oat and wheat 14-3-3 proteins formed closer phylogenetic clusters than those from *A. thaliana* or rice, indicating a closer evolutionary relationship between these two species.

**Figure 3:**
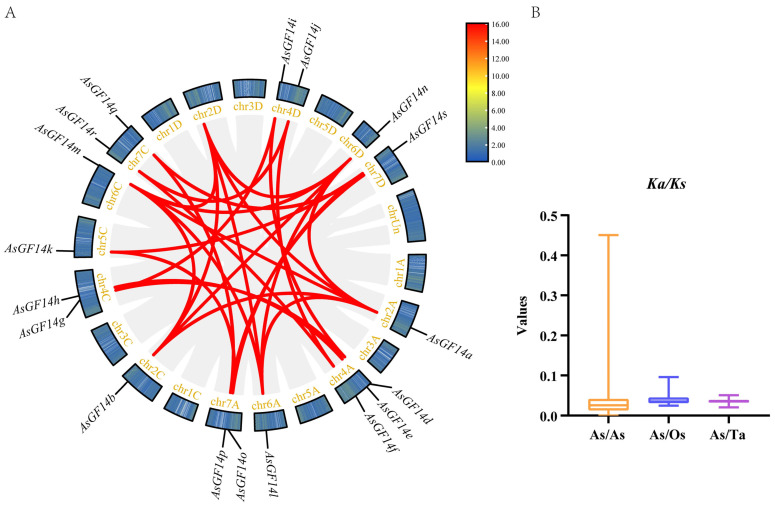
Duplication and evolutionary analysis of the *AsGF14* gene family. (**A**) Intra-genomic synteny and duplication events. Gray lines represent systemic blocks within the *A. sativa* genome; red lines highlight systemic paralogous *AsGF14* gene pairs. (**B**) *Ka/Ks* values for homologous gene pairs. As/As refers to paralogous pairs within *A. sativa*; As/Os refers to orthologous pairs between *A. sativa* and *O. sativa*; and As/Ta refers to orthologous pairs between *A. sativa* and *T. aestivum*.

To investigate the genomic mechanisms driving the expansion of the *AsGF14* gene family in oat, we performed a genome-wide synteny analysis. A total of 20 *AsGF14*-*AsGF14* syntenic pairs were identified, involving 18 of the 19 *AsGF14* genes (Figure 3A). All syntenic *AsGF14* genes were associated with segmental duplications, with no evidence of tandem duplication. The ε group contained three syntenic pairs, whereas the non-ε group comprised the remaining 17. These segmental duplications represent the primary driver of the expansion of non-ε *AsGF14* genes, suggesting that non-ε members have undergone functional diversification.

**Figure 4:**
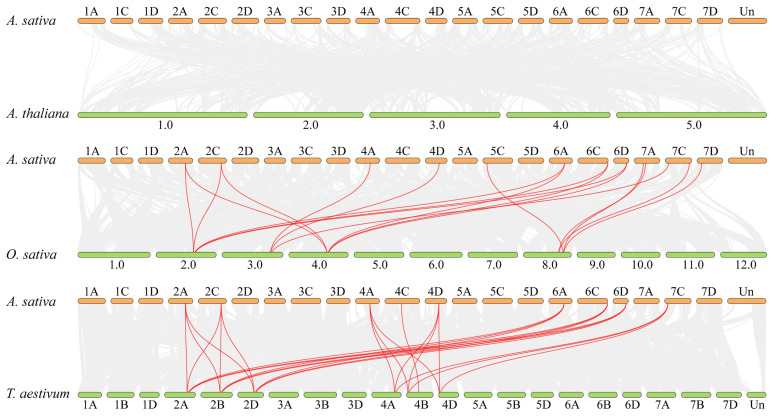
Syntenic relationships between the *14-3-3* genes of *A. sativa* and three other species (*A. thaliana*, *O. sativa* and *T. aestivum*). Gray lines represent syntenic blocks within the genomes, and red lines highlight collinear *14-3-3* gene pairs.

Comparative synteny analysis revealed extensive synteny between oat and three other species (Figure 4). Specifically, *AsGF14* genes formed 25 syntenic pairs with wheat orthologs, with eight *AsGF14* members (*AsGF14a*, *AsGF14b*, *AsGF14f*, *AsGF14j*, *AsGF14L*, *AsGF14m*, *AsGF14n*, and *AsGF14r*) each syntenic with three wheat orthologs (Table S2). Thirteen *AsGF14* genes were syntenic with rice orthologs, forming 18 syntenic pairs; notably, five of these (*AsGF14a*, *AsGF14b*, *AsGF14L*, *AsGF14m*, and *AsGF14n*) each paired with two rice orthologs (Table S3). No synteny was detected between oat and *A. thaliana 14-3-3* genes. The *Ka/Ks* ratios for the oat–wheat syntenic pairs ranged from 0.0206 to 0.0509 (Figure 3B, Table S4), while those for oat–rice pairs ranged from 0.0249 to 0.0962 (Figure 3B, Table S5). All values were substantially less than 1, indicating that these orthologous *14-3-3* gene pairs have undergone strong purifying selection during evolution. Divergence times were estimated from *Ks* values using the molecular clock model. The average divergence time between *A. sativa* and *T. aestivum* was approximately 37.24 million years ago (Mya) (Table S4), and between *A. sativa* and *O. sativa*, approximately 44.49 Mya (Table S5).

Selection pressures on duplicated *AsGF14*-*AsGF14* gene pairs were assessed by calculating *Ka/Ks* ratios (Figure 3B, Table S6). One syntenic pair (*AsGF14d*/*AsGF14i*), which exhibited a *Ks* value of zero, was excluded from further selection pressure analysis because a reliable *Ka/Ks* ratio could not be calculated. For all remaining syntenic pairs, *Ka/Ks* ratios were substantially less than 1, indicating that the *AsGF14* gene family has predominantly undergone purifying selection. Notably, four gene pairs displayed *Ka/Ks* values of zero, suggesting strong functional constraint and no nonsynonymous substitutions since duplication. Interestingly, the ε group exhibited a higher average *Ka/Ks* ratio compared to the non-ε group, implying relatively relaxed selection within this group. The average divergence time for duplicated *AsGF14* pairs was approximately 22.58 Mya (Table S6).

### 2.3. Gene Structure and Conserved Motif Analysis of AsGF14 Genes

**Figure 5:**
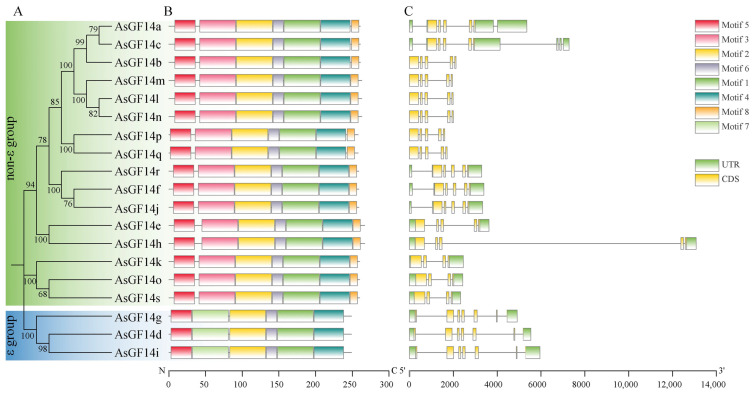
Phylogeny, conserved motifs and exon–intron structures of *AsGF14* genes. (**A**) Phylogenetic analysis of AsGF14 proteins. (**B**) Motif analysis of AsGF14 proteins. (**C**) Exon–intron structures of *AsGF14* genes.

To gain insights into the structural and functional divergence of the *AsGF14* family, we integrated phylogenetic classification analysis with gene structure and conserved characterization (Figure 5, Figure S3). Conserved motif analysis revealed that all members of the ε group contained six conserved motifs: motifs 1, 2, 4, 5, 6, and 7. In contrast, AsGF14 proteins in the non-ε group harbored seven motifs: motifs 1, 2, 3, 4, 5, 6, and 8. Notably, motif 3 and motif 8 were exclusively present in the non-ε group, whereas motif 7 was exclusively present in the ε group, suggesting potential functional specialization. Gene structure analysis revealed that ε group genes contained seven exons, the most among *AsGF14* genes. In comparison, non-ε group genes contained four or five exons: *AsGF14k*, *AsGF14o*, and *AsGF14s* had four exons, while the remaining 13 possessed five. The distinct motif architectures and exon–intron organizations of *AsGF14* genes in these two phylogenetic groups indicate evolutionary and functional divergence.

### 2.4. Cis-Acting Elements in the Promoter Regions of AsGF14 Genes

**Figure 6:**
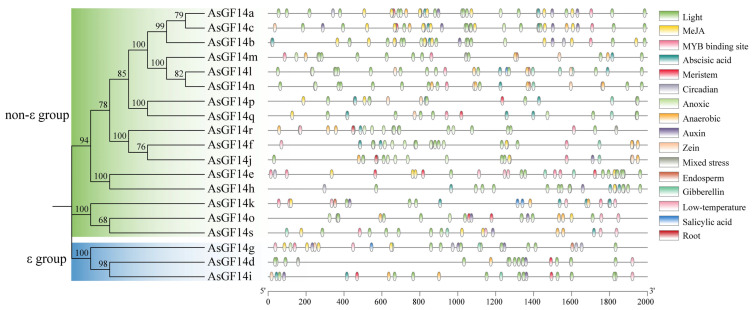
*Cis*-acting element analysis in the promoter of *AsGF14* genes.

Analysis of *cis*-acting elements in *AsGF14* promoters identified 16 distinct types associated with hormone responses, environmental stress, and growth and development (Figure 6, Figure S4). Abscisic acid- and light-responsive elements were present in all promoters. Fourteen genes contained anoxic stress-responsive and MYB-binding site elements; 13 harbored anaerobic stress-responsive and MeJA-responsive elements; 11 contained auxin-responsive and low-temperature-responsive elements; and 10 harbored gibberellin-responsive and zein metabolism-regulation elements. Additionally, eight genes contained meristem-specific elements; five contained circadian control and endosperm-specific elements; and three contained elements linked to mixed stress, root specificity, and salicylic acid responsiveness. The total number of *cis*-elements varied considerably among promoters, ranging from 20 in *AsGF14m* to 45 in *AsGF14c*.

### 2.5. Expression Patterns of AsGF14 Genes Under Drought Stress

**Figure 7:**
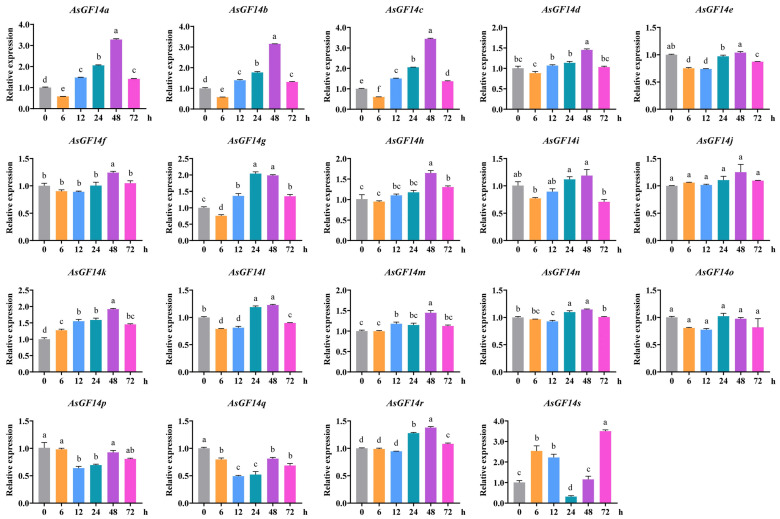
Relative expression of *AsGF14* genes under drought stress. Error bars show mean ± SEM of three independent experiments. Statistical significance was determined using one-way ANOVA with Tukey’s test. Different lowercase letters indicate significant differences (*p* < 0.05).

To characterize the expression patterns of *AsGF14* genes under drought stress, we profiled *AsGF14* gene expression in root tissues at six time points (0, 6, 12, 24, 48, and 72 h) following drought treatment (Figure 7). Six genes (*AsGF14d*, *AsGF14f*, *AsGF14h*, *AsGF14k*, *AsGF14m*, and *AsGF14r*) were significantly upregulated at one or more time points. Notably, *AsGF14k* exhibited sustained upregulation throughout the treatment period, peaking at 48 h. Conversely, three genes (*AsGF14i*, *AsGF14j*, and *AsGF14o*) showed no significant differential expression. Three others (*AsGF14e*, *AsGF14p*, and *AsGF14q*) were significantly downregulated at specific time points. The remaining seven genes (*AsGF14a*, *AsGF14b*, *AsGF14c*, *AsGF14g*, *AsGF14L*, *AsGF14n*, and *AsGF14s*) displayed dynamic and variable expression patterns, with transient upregulation or downregulation at specific time points. This differential expression suggests functional diversification among *AsGF14* genes during drought adaptation.

### 2.6. Subcellular Localization of the AsGF14k Protein

**Figure 8:**
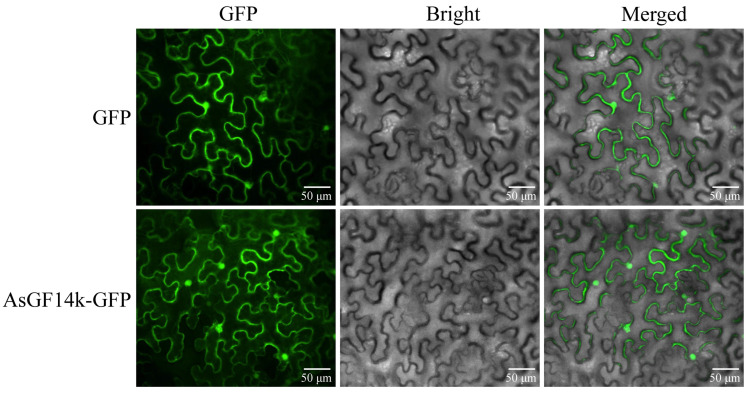
Subcellular localization of AsGF14k. Bars, 50 μm.

Given that *AsGF14k* was continuously and significantly upregulated under drought stress, we investigated its subcellular localization by transiently expressing an AsGF14k-GFP fusion protein in *Nicotiana benthamiana* leaves. Confocal microscopy revealed that the AsGF14k-GFP signal localized to multiple subcellular compartments, including the nucleus and cytoplasm (Figure 8).

### 2.7. Assessment of AsGF14k Homodimerization by Yeast Two-Hybrid (Y2H)

**Figure 9:**
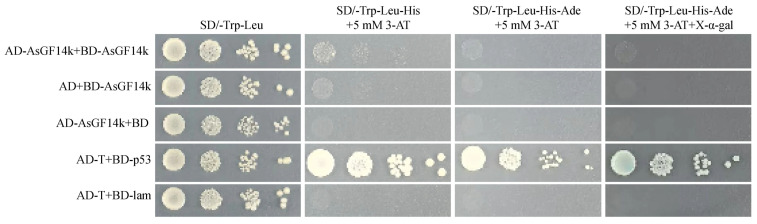
Y2H analysis of AsGF14k protein interactions. AD-T + BD-p53 and AD-T + BD-lam served as the positive and negative controls, respectively.

To determine whether AsGF14k functions as a homodimer, we performed a Y2H assay. The BD-AsGF14k fusion protein exhibited transcriptional autoactivation activity, which was effectively suppressed by 5 mM 3-amino-1,2,4-triazole (3-AT) (Figure S5). Yeast co-transformed with AD-AsGF14k and BD-AsGF14k grew normally on SD/-Trp-Leu medium but failed to grow on selective media (SD/-Trp-Leu-His and SD/-Trp-Leu-His-Ade) supplemented with 5 mM 3-AT and showed no blue coloration on SD/-Trp-Leu-His-Ade plates containing X-α-gal (Figure 9). These results indicate that AsGF14k does not self-interact, suggesting it likely does not function as a homodimer.

## 3. Discussion

The nomenclature of the *14-3-3* gene family exhibits considerable variation across plant species. For instance, members are designated GRF followed by Arabic numerals in *Medicago sativa* (*MsGRF1–MsGRF66*) [33] and *Camellia sinensis* (*CsGRF1–CsGRF26*) [34], or by lowercase letters in *Arachis hypogaea* (*AhGRFa–AhGRFv*) [35]. In other species, the prefix “14-3-3” is combined with a letter, as in *Olyra latifolia* (*Ol14-3-3a–Ol14-3-3h*) [36], or with Roman numerals, as in *Manihot esculenta* (*Me14-3-3I–Me14-3-3XVI*) [8]. Alternatively, the prefix GF14 is used in *O. sativa* (*OsGF14a–OsGF14h*) [12] and *Malus domestica* (*MdGF14a–MdGF14r*) [37]. In this study, to maintain consistency with rice nomenclature, we adopted the GF14-based system for oat *14-3-3* genes, designating them *AsGF14a* to *AsGF14s*. All AsGF14 proteins exhibited a pI below 7, consistent with their classification as acidic proteins. Notably, ε group members displayed higher pI values than non-ε group members, suggesting distinct electrostatic properties between groups. Regarding protein stability, an instability index above 40 typically predicts an unstable protein [38]. All ε group AsGF14 proteins exhibited instability indices below 40, whereas only three non-ε group members (AsGF14k, AsGF14o, and AsGF14s) fell below this threshold, indicating that ε group AsGF14 proteins are likely more stable than their non-ε isoforms.

Phylogenetic analysis classifies 14-3-3 proteins into distinct groups, providing insights into their structural features, evolutionary history, and functional conservation or divergence. The 14-3-3 proteins in *A. thaliana* and rice are classified into ε and non-ε groups, with the ε group comprising AtGRF9–AtGRF13 in *A. thaliana* and OsGF14g–OsGF14h in rice [1,12]. Accordingly, we applied this conserved phylogenetic framework to classify oat AsGF14 proteins, identifying three ε group members. Moreover, ε group *14-3-3* genes consistently contain more exons than non-ε genes in multiple plant species, including *O. sativa* [1], *Solanum tuberosum* [39] and *M. esculenta* [8]. Consistent with this pattern, all three ε group *AsGF14* genes contain seven exons, whereas non-ε group members possess four or five. These differences suggest evolutionary divergence in gene structure between groups. In contrast, non-ε group AsGF14 proteins contain more conserved motifs than ε group proteins. Specifically, motif 7 is unique to the N-terminus of ε-group proteins, whereas motifs 3 and 8 are specific to the N- and C-termini of non-ε proteins, respectively. Previous studies suggest that the C-terminal region of 14-3-3 proteins may be involved in target protein specificity, whereas the N-terminal region contributes to dimerization [14]. Therefore, we speculate that these group-specific motifs may contribute to functional divergence between ε and non-ε AsGF14 proteins. *Cis*-regulatory element analysis further revealed distinct compositional patterns between groups: all ε group members contained *cis*-elements responsive to abscisic acid, auxin, light, and low temperature, whereas non-ε group members consistently harbored only abscisic acid– and light-responsive elements. *Cis*-acting elements associated with drought stress responses primarily include abscisic acid-responsive elements (ABREs), dehydration-responsive elements (DREs), and MYB-binding sites (MBS) [40]. In this study, ABREs were identified in the promoter regions of all six drought-upregulated *AsGF14* genes (*AsGF14d*, *AsGF14f*, *AsGF14h*, *AsGF14k*, *AsGF14m*, and *AsGF14r*), with copy numbers ranging from one to six. Furthermore, MBS elements were also detected in the promoters of *AsGF14f*, *AsGF14k*, *AsGF14m*, and *AsGF14r*. These findings suggest that these *cis*-elements may play a role in oat drought adaptation (Figure S4). Collectively, these divergent features in gene structure, conserved motifs, and regulatory profiles are consistent with the phylogenetic clustering of *AsGF14* genes, suggesting evolutionary divergence and likely functional specialization between groups.

Gene duplication is a major driver of gene family expansion and functional diversification in plant evolution [41,42]. Hexaploid oat has undergone a whole-genome duplication (WGD) event during its evolutionary history [43]. Our analysis revealed that the *AsGF14* gene family expanded primarily through segmental duplications, with no evidence of tandem duplications. This indicates that WGD and segmental duplications were the predominant mechanisms underlying *AsGF14* gene family expansion. To assess selective pressures acting on duplicated genes, we calculated the *Ka/Ks* ratio, a widely used metric for evaluating evolutionary constraints [44]. *Ka/Ks* ratios below 1 indicate purifying selection [45]. All 19 syntenic paralogous pairs of *AsGF14* genes exhibited *Ka/Ks* ratios less than 1. Similarly, *Ka/Ks* ratios between *AsGF14* genes and their orthologs in rice and wheat were also below 1. Notably, homoeologous pairs within the wheat *14-3-3* gene family have likewise been reported to exhibit *Ka/Ks* ratios below 1 [22]. Collectively, these results demonstrate that the *14-3-3* gene family has been under strong purifying selection and displays a high degree of functional conservation across oat, rice, and wheat.

14-3-3 proteins are crucial regulators of plant metabolism and signal transduction, governing growth, development, and stress adaptation, particularly to drought. For instance, in rice, OsGF14f positively modulates drought tolerance by interacting with the transcription factor OsbZIP23 [46]. In wheat, TaGF14b enhances drought tolerance through interaction with TaABF2 to potentiate ABA signaling [47]. Conversely, silencing *Hv14-3-3A* in barley increases stomatal density, reduces water-use efficiency, and heightens drought sensitivity [24]. Similar to the drought-induced expression of *OsGF14f*, *TaGF14b*, and *Hv14-3-3A*, we observed transcriptional upregulation of six *AsGF14* genes under drought stress, suggesting that transcriptional activation of specific *14-3-3* isoforms constitutes a conserved drought response mechanism across cereal crops. Notably, *AsGF14k* exhibited significant and sustained upregulation, highlighting it as a promising candidate for drought tolerance in oat. However, transcriptional induction alone does not conclusively demonstrate functional involvement. Therefore, future studies will focus on functional validation through genetic transformation or gene silencing to determine the specific roles of *AsGF14k* in drought tolerance. Furthermore, plant roots are complex organs composed of diverse cell types with distinct functions. Although qRT-PCR reveals global expression trends of *AsGF14* genes, these represent tissue-averaged signals. Recent studies indicate that stress responses can be highly cell-type-specific [11]. Consequently, the cell-type-specific expression of *AsGF14* genes remains to be elucidated.

14-3-3 proteins modulate the activity, stability, and subcellular localization of target proteins via direct binding and are broadly distributed, consistent with their diverse cellular roles [14]. For instance, ApGRF6-2 has been detected in both the cytoplasm and nucleus [4], whereas PbGRF8 and PbGRF18 localize to the cytoplasm and plasma membrane; moreover, PbGRF11 and MsGRF2 have been observed in the cytoplasm, plasma membrane, and nucleus [5,33]. In this study, AsGF14k localized to both the cytoplasm and nucleus, suggesting potential involvement in cytoplasmic signaling and nuclear transcriptional regulation. This dual localization may enable the transmission of drought signals from the cytoplasm to the nucleus, potentially influencing transcription factor activity. However, whether this reflects active nucleocytoplasmic shuttling under drought stress requires further investigation. 14-3-3 proteins typically function as homodimers or heterodimers. For instance, the apple 14-3-3 protein MdGRF10 forms a homodimer to interact with MdASMT1, thereby enhancing salt tolerance [19]. In contrast, in vitro biochemical analyses have shown that the peanut 14-3-3 protein AhGRFi is incapable of homodimer formation [35]. Consistent with this, our Y2H assay revealed that AsGF14k exhibits no self-interaction, suggesting it is unlikely to function as a homodimer. This finding raises the possibility that AsGF14k functions as a monomer or preferentially forms heterodimers with other partners. Future studies should focus on identifying these interacting partners and determining whether post-translational modifications regulate AsGF14k activity under drought conditions.

## 4. Materials and Methods

### 4.1. Genome-Wide Identification and Characterization of the 14-3-3 Gene Family in Oat

The genome data for *A. sativa* were obtained from the GrainGenes database (https://wheat.pw.usda.gov/GG3/, accessed on 22 October 2024) [48]. To identify 14-3-3 proteins, previously characterized 14-3-3 sequences from *A. thaliana* (13 members) (Table S7) [49] and *O. sativa* (8 members) (Table S7) [12] were used as queries in BLASTP searches against the *A. sativa* protein dataset, with an E-value cutoff of 1 × 10^−5^. Additionally, the Hidden Markov Model (HMM) profile of the 14-3-3 domain (PF00244) was employed to scan the *A. sativa* genome [50]. The overlapping candidates from the BLASTP and HMM searches were filtered to remove incomplete gene models, retaining the longest transcript isoform per locus. The presence of the conserved 14-3-3 domain in all candidate proteins was further confirmed using the NCBI-CDD (https://www.ncbi.nlm.nih.gov/Structure/cdd/wrpsb.cgi, accessed on 18 May 2025), Pfam (http://pfam.xfam.org/, accessed on 18 May 2025), and SMART (https://smart.embl.de/, accessed on 18 May 2025) databases. Only sequences containing a complete 14-3-3 domain were considered members of the oat *14-3-3* gene family. The physicochemical properties of the AsGF14 proteins were analyzed using TBtools v2.423 [51].

### 4.2. Chromosomal Location, Gene Structure, Conserved Motif, and Cis-Acting Analysis

The chromosomal locations and exon–intron structures of the *AsGF14* genes were determined using TBtools based on the genome annotation of *A. sativa*. Conserved protein motifs were identified by submitting the AsGF14 protein sequences to the MEME web server, with the maximum number of motifs set to 8 and the remaining parameters at default settings [52]. To analyze *cis*-acting regulatory elements, the 2000 bp promoter region upstream of the start codon was extracted for each *AsGF14* gene and analyzed using the PlantCARE database (https://bioinformatics.psb.ugent.be/webtools/plantcare/html/, accessed on 27 May 2025) [53]. The chromosomal locations, gene structures, conserved motifs, and *cis*-acting elements were then integrated and visualized using TBtools.

### 4.3. Sequence Alignment, 3D Structure Prediction, and Phylogenetic Tree Construction

The amino acid sequences of 14-3-3 proteins in *A. sativa* were analyzed using DNAMAN 7 software. The 3D structures of these proteins were predicted with AlphaFold 3 and visualized using PyMOL v3.0.3 [54]. A phylogenetic tree was constructed from the aligned amino acid sequences of the *14-3-3* gene family across *A. thaliana*, *O. sativa*, *T. aestivum* (Table S7), and *A. sativa*. Multiple sequence alignment was performed with ClustalW in MEGA v7.0. A maximum-likelihood tree was generated with 1,000 bootstrap replicates and visualized using iTOL (https://itol.embl.de/, accessed on 29 May 2025) [55].

### 4.4. Synteny Analysis and Selection Pressure of AsGF14 Genes

Synteny analysis between *A. sativa* and three other species (*A. thaliana*, *O. sativa*, and *T. aestivum*) was performed using the One Step MCScanX module in TBtools. Subsequently, the non-synonymous (*Ka*) and synonymous (*Ks*) substitution rates for all syntenic gene pairs were calculated using the Simple Ka/Ks Calculator in TBtools. *Ka/Ks* ratios >1, <1, and =1 were considered indicative of positive, purifying, and neutral selection, respectively [45]. The divergence time was estimated using the formula T = *Ks*/(2λ), where λ represents the synonymous substitution rate per site per year. Following previous studies in grasses, we adopted λ = 6.5 × 10^−9^ [56].

### 4.5. Plant Materials

Oat seeds (cv. Pinyan No. 8), provided by the Center for Agricultural Genetic Resources Research at Shanxi Agricultural University, were surface-sterilized with 1.0% sodium hypochlorite for 20 min and rinsed three times with sterile distilled water [57]. After germination in Petri dishes, the seedlings were transferred to a modified Hoagland nutrient solution and grown in a greenhouse under a 16 h light/8 h dark photoperiod at 25 °C with a light intensity of 250 μmol·m^−2^·s^−1^. At the one-leaf stage, drought stress was simulated by adding polyethylene glycol 6000 (PEG 6000) to the nutrient solution to a final concentration of 15% (*w*/*v*) [58]. Root samples were collected at 0, 6, 12, 24, 48, and 72 h post-treatment, with three biological replicates for each time point.

### 4.6. qRT-PCR Analysis

Total RNA was extracted from each sample using the DP432 RNAprep Pure Plant Kit (Tiangen, Beijing, China). First-strand cDNA was synthesized with the HiScript IV All-in-One Ultra RT SuperMix (Vazyme, Nanjing, China). qRT-PCR was performed using FastReal qPCR PreMix (Tiangen, China). The *AsActin* gene was used as an internal reference. Relative expression levels were calculated using the 2^−ΔΔCt^ method and are presented as the mean ± SEM of three technical replicates [59]. All primer sequences are listed in Table S8.

### 4.7. Subcellular Location

The coding sequence of *AsGF14k* (excluding the stop codon) was cloned into the *pCAMBIA1302* vector to generate a C-terminal GFP fusion. The construct was transformed into *Agrobacterium* strain GV3101 and transiently expressed in *N. benthamiana* leaves via agroinfiltration. The empty *pCAMBIA1302-GFP* vector served as a control [60]. Fluorescence signals were visualized using a spinning disk confocal laser-scanning microscope (UltraView VoX, PerkinElmer, Waltham, MA, USA). Each experiment was performed with three biological replicates. GFP was excited using a 488 nm solid-state laser (UltraView VoX, PerkinElmer, Waltham, MA, USA), and emission signals were collected through a 525/50 nm band-pass filter (emission filter wheel, position 2).

### 4.8. Y2H Assay

The *AsGF14k* cDNA was cloned into the *pGADT7* (AD) and *pGBKT7* (BD) vectors. The constructs were co-transformed into the yeast strain AH109 [61]. Transformants were initially selected on SD/-Leu/-Trp medium. Protein interactions were assessed by growth on SD/-Leu/-Trp/-His and SD/-Leu/-Trp/-His/-Ade media supplemented with 5 mM 3-AT and by blue colony formation on plates containing X-α-gal. AD-T + BD-p53 and AD-T + BD-lam were used as the positive and negative controls, respectively. Primers are listed in Table S8.

## Figures and Tables

**Table 1:** Detailed in formation of *AsGF14* genes in oat.

Gene Name	Gene ID	Amino Acid Number	Molecular Weight (kDa)	pI	Instability Index	GRAVY	14-3-3 Domain	Group
*Asgf14a*	AVESA.00010b.r2.2AG0225100.1	260	29.61	4.72	51.42	−0.483	9–253	non-ε group
*AsGF14b*	AVESA.00010b.r2.2CG0304460.1	260	29.56	4.72	48.94	−0.460	9–253	non-ε group
*AsGF14c*	AVESA.00010b.r2.2DG0378320.1	260	29.59	4.72	50.35	−0.473	9–253	non-ε group
*AsGF14d*	AVESA.00010b.r2.4AG0574560.1	248	28.20	5.03	36.09	−0.317	10–229	ε group
*AsGF14e*	AVESA.00010b.r2.4AG0578030.1	266	29.25	4.76	51.93	−0.455	8–248	non-ε group
*AsGF14f*	AVESA.00010b.r2.4AG0602980.1	258	29.01	4.82	42.52	−0.443	7–250	non-ε group
*AsGF14g*	AVESA.00010b.r2.4CG1298730.1	248	28.17	5.09	39.81	−0.296	10–229	ε group
*AsGF14h*	AVESA.00010b.r2.4CG1301940.1	266	29.34	4.80	51.20	−0.471	8–248	non-ε group
*AsGF14i*	AVESA.00010b.r2.4DG0718860.1	248	28.22	5.10	34.54	−0.295	10–229	ε group
*AsGF14j*	AVESA.00010b.r2.4DG0746960.1	258	29.01	4.82	42.52	−0.443	7–250	non-ε group
*AsGF14k*	AVESA.00010b.r2.5CG0924170.1	259	28.69	4.80	37.04	−0.319	8–244	non-ε group
*AsGF14L*	AVESA.00010b.r2.6AG1038270.1	262	29.66	4.70	49.39	−0.502	9–252	non-ε group
*AsGF14m*	AVESA.00010b.r2.6CG1076180.1	262	29.67	4.66	49.83	−0.503	9–252	non-ε group
*AsGF14n*	AVESA.00010b.r2.6DG1161890.1	262	29.66	4.70	49.39	−0.502	9–252	non-ε group
*AsGF14o*	AVESA.00010b.r2.7AG1224470.1	259	28.69	4.80	37.91	−0.319	8–244	non-ε group
*AsGF14p*	AVESA.00010b.r2.7AG1227370.1	257	28.88	4.79	45.01	−0.420	3–239	non-ε group
*AsGF14q*	AVESA.00010b.r2.7CG0661980.1	257	28.88	4.79	45.01	−0.420	3–239	non-ε group
*AsGF14r*	AVESA.00010b.r2.7CG0698850.1	258	28.99	4.82	41.29	−0.433	7–250	non-ε group
*AsGF14s*	AVESA.00010b.r2.7DG1383830.1	259	28.69	4.80	37.91	−0.319	8–244	non-ε group

## Data Availability

The original contributions presented in the study are included in the article and Supplementary Materials, further inquiries can be directed to the corresponding author.

## Supplementary Materials

The following supporting information can be downloaded at: https://www.mdpi.com/article/10.3390/plants15091280/s1. Figure S1: Multiple amino acid sequence alignment of AsGF14 proteins. The blue frame indicates the 14-3-3 domain; Figure S2: 3D structure prediction analysis of AsGF14 proteins; Figure S3: Conserved motifs of AsGF14 proteins; Figure S4: Number of *cis*-acting regulatory elements in the promoters of *AsGF14* genes; Figure S5: Self-activation and suppression assays of the AsGF14k protein in Y2H systems. AD-T + BD-p53 and AD-T + BD-lam served as the positive and negative controls, respectively; Table S1: Protein and coding sequences of the *AsGF14* gene family in oat; Table S2: Orthologous pairs of *14-3-3* genes between *A. sativa* and *T. aestivum*; Table S3: Orthologous pairs of *14-3-3* genes between *A. sativa* and *O. sativa*; Table S4: *Ka/Ks* ratios of *14-3-3* orthologous genes between *A. sativa* and *T. aestivum*; Table S5: *Ka/Ks* ratios of *14-3-3* orthologous genes between *A. sativa* and *O. sativa*; Table S6: Selection pressure on *AsGF14* genes in oat; Table S7: Protein sequences of the *14-3-3* gene family in *A. thaliana*, *O. sativa*, and *T. aestivum*; Table S8: Primer sequences used in this study for *AsGF14* genes.
