# Supplementary material for: Genome-Wide Identification and Characterization of the 14-3-3 Gene Family in Avena sativa
Source: Plants (Basel). 2026 Apr 22;15(9):1280. doi: 10.3390/plants15091280 (PMC13165189; doi:10.3390/plants15091280)
Supplement: Supplementary file 1 [file plants-15-01280-s001.zip › plants-4249878-figures.pdf]

Supplementary Material

Supplementary Figures

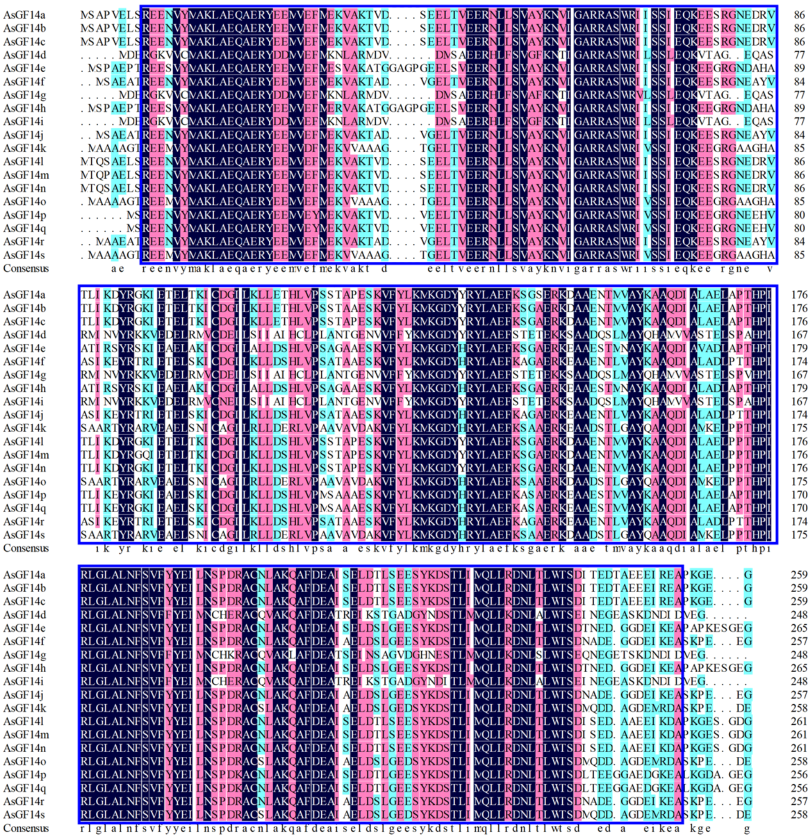

Figure S1. Multiple amino acid sequence alignment of AsGF14 proteins. The blue frame indicated the 14-3-3 domain.

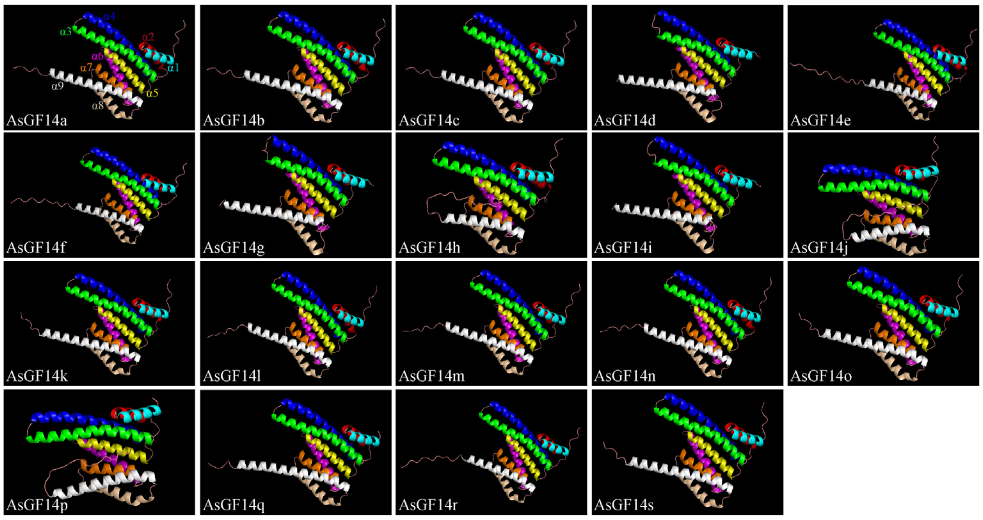

Figure S2. 3D structure prediction analysis of AsGF14 proteins.

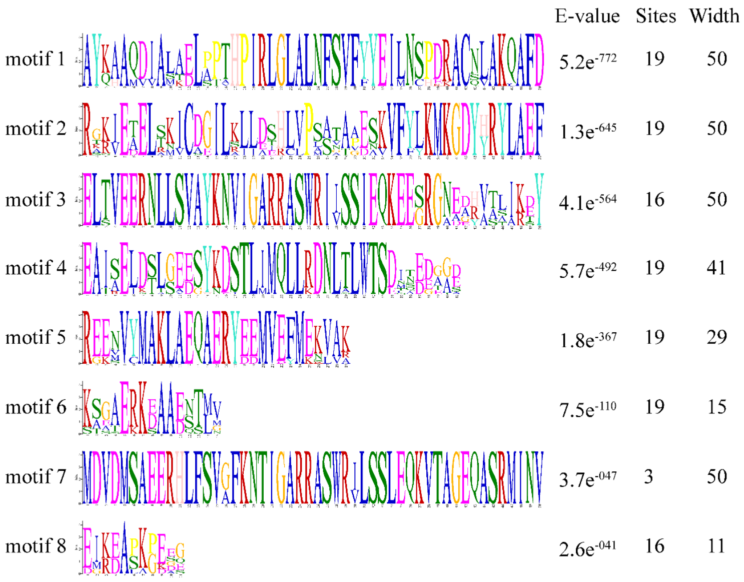

Figure S3. Conserved motifs of AsGF14 proteins.

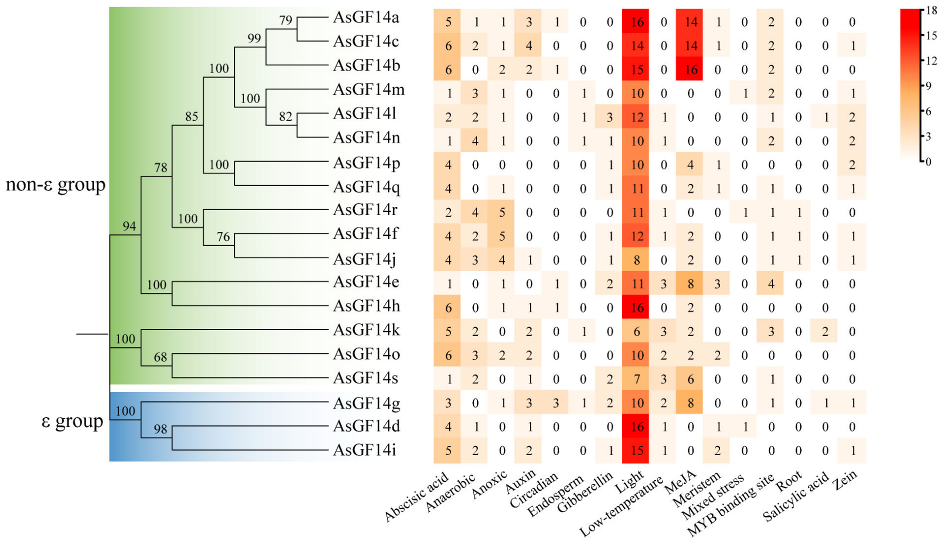

Figure S4. Number of *cis*-acting regulatory elements in the promoters of *AsGF14* genes.

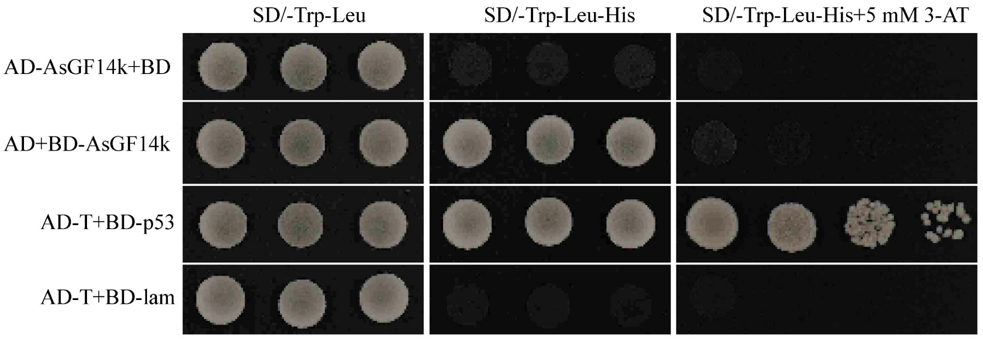

Figure S5. Self-activation and suppression assays of the AsGF14k protein in Y2H systems. AD-T + BD-p53 and AD-T + BD-lam served as the positive and negative controls, respectively.
